# Testosterone Deficiency Induces Changes of the Transcriptomes of Visceral Adipose Tissue in Miniature Pigs Fed a High-Fat and High-Cholesterol Diet

**DOI:** 10.3390/ijms17122125

**Published:** 2016-12-16

**Authors:** Lifan Zhang, Yueqin Cai, Shengjuan Wei, Yun Ling, Liang Zhu, Dongfeng Li, Zhaowei Cai

**Affiliations:** 1 College of Animal Science and Technology, Nanjing Agricultural University, Nanjing 210095, China; sjwei@njau.edu.cn (S.W.); lidongfeng@njau.edu.cn (D.L.); 2 Laboratory Animal Research Center, Zhejiang Chinese Medical University, Hangzhou 310053, China; cyq810@hotmail.com (Y.C.); lyun1983@163.com (Y.L.); tozhuliang@126.com (L.Z.)

**Keywords:** testosterone deficiency, visceral adipose tissue, inflammatory response, miniature pigs, RNA-Seq

## Abstract

Testosterone deficiency causes fat deposition, particularly in visceral fat, and its replacement might reverse fat accumulation, however, the underlying mechanisms of such processes under diet-induced adiposity are largely unknown. To gain insights into the genome-wide role of androgen on visceral adipose tissue (VAT), RNA-Seq was used to investigate testosterone deficiency induced changes of VAT in miniature pigs fed a high-fat and high-cholesterol (HFC) diet among intact male pigs (IM), castrated male pigs (CM), and castrated male pigs with testosterone replacement (CMT) treatments. The results showed that testosterone deficiency significantly increased VAT deposition and serum leptin concentrations. Moreover, a total of 1732 differentially expressed genes (DEGs) were identified between any two groups. Compared with gene expression profiles in IM and CMT pigs, upregulated genes in CM pigs, i.e., *LOC100520753* (*CD68*), *LCN2*, *EMR1*, *S100A9*, *NCF1* (*p47phox*), and *LEP*, were mainly involved in inflammatory response, oxidation-reduction process, and lipid metabolic process, while downregulated genes in CM pigs, i.e., *ABHD5*, *SPP1*, and *GAS6*, were focused on cell differentiation and cell adhesion. Taken together, our study demonstrates that testosterone deficiency alters the expression of numerous genes involved in key biological processes of VAT accumulation under HFC diet and provides a novel genome-wide view on the role of androgen on VAT deposition under HFC diet, thus improving our understanding of the molecular mechanisms involved in VAT changes induced by testosterone deficiency.

## 1. Introduction

Visceral adiposity represents excess visceral fat tissue (VAT) deposition in body, which is associated with several critical human diseases such as type 2 diabetes, metabolic syndrome and cardiovascular disease, suggesting visceral adiposity is a high disease risk factor in the life. As we know, excessive nutrients intake from the daily diet, such as high-fat and high-cholesterol (HFC) diet, significantly increases VAT accumulation [1,2]. On the other hand, sex hormone levels might also contribute to VAT deposition. For example, low testosterone levels result in excess VAT accumulation or waist circumference in men, thus increasing visceral adiposity [3,4,5], indicating that diet and sex hormone could play important roles on visceral adiposity. In some cases, more severe testosterone deficiency and wider waist circumference were observed in obese or diabetic individuals with aging or hypogonadal state [3], illustrating that testosterone deficiency in obese or diabetic individuals might further aggravate the extent of visceral adiposity.

Conversely, to avoid visceral adiposity induced by testosterone deficiency, a feasible strategy is testosterone replacement, which resulted in a significant decrease in body mass index, fat mass, and regional fat distribution in hypogonadal and aging men [6,7]. Furthermore, testosterone treatment reduced waist circumference, waist/hip ratio, visceral fat mass and metabolic syndrome mainly caused by visceral adiposity in obese hypogonadal men [8,9], strongly suggesting that testosterone replacement might be used for visceral adiposity treatment in men caused by testosterone deficiency. Most recently, a review pointed out that appropriate testosterone therapy in obese men with testosterone deficiency is a novel medical approach to treat obesity [10]. In other animal models, testosterone replacement decreased visceral fat weight and size in rabbits with high-fat (HFD) diet [11] and visceral fat size in hypogonadal and aged male rats [12]. Therefore, these data clearly show that testosterone replacement might be considered as a safe and effective therapy for obesity in men or animals with testosterone deficiency [13].

In general, pig model has been widely used for studying the changes induced by testosterone deficiency because it was shown to have similar characteristics concerning obesity and metabolic syndrome to human [14,15,16]. Therefore, by using pig model, our previous study discovered that castration-induced testosterone deficiency resulted in significant changes of miRNAs in subcutaneous adipose tissue, which were associated with fatty acid metabolism by acting on their target genes [17]. Moreover, we also found that testosterone deficiency caused severe hypercholesterolemia and hepatic steatosis in pigs fed a HFC diet and could be reversed by testosterone replacement therapy [18,19]. However, thus far, little is known about the underlying mechanisms of changes in VAT induced by testosterone deficiency under HFC diet, particularly in alterations of gene expression and regulation in VAT caused by testosterone changes. To shed light on this issue, we studied the characteristics of gene expression in VAT under the changes of testosterone on pigs fed a HFC diet by RNA-Seq technology, thus providing novel genome-wide insights in mechanisms involved in androgen roles on VAT metabolism.

## 2. Results

### 2.1. Effects of Castration and Testosterone Treatment on VAT Fat and Serum Leptin Levels

**Figure 1:**
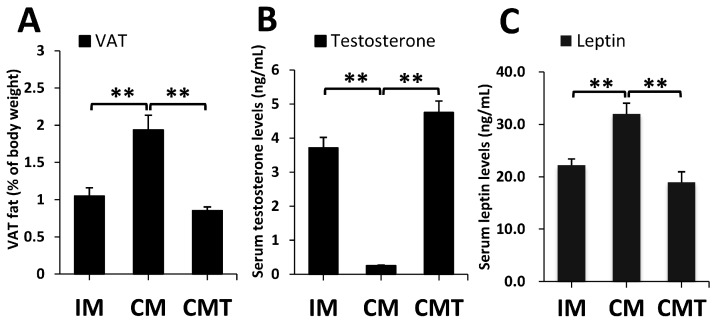
Effects of castration and testosterone treatment on visceral adipose tissue (VAT) fat, serum testosterone and leptin levels: (**A**) VAT fat percentage of body weight; (**B**) serum testosterone levels; and (**C**) leptin levels. IM: intact male pigs fed a high-fat and high-cholesterol (HFC) diet; CM: castrated male pigs fed a HFC diet; CMT: castrated pigs with testosterone replacement fed a HFC diet. Data were represented as means ± SEMs, *n* = 4 per group. ** *p* < 0.01.

Castration significantly enhanced VAT fat, while testosterone treatment significantly decreased VAT fat, with no significant difference between IM and CMT pig groups (Figure 1A). By contrast, testosterone levels significantly decreased in CM pigs but increased in CMT pigs (Figure 1B). Consistent with the changes of VAT fat, serum leptin levels significantly increased in CM pigs but decreased in CMT pigs (Figure 1C).

### 2.2. Characteristics of RNA-Seq

A total of approximately 26.81, 24.65, and 23.87 million high quality clean reads were obtained from IM, CM, and CMT pigs, respectively. More than 81% of reads could be mapped to the pig reference genome 10.2. Furthermore, more than 77% of these mapped reads aligned with unique genes (Table 1). Finally, 16,836 mRNAs were found to be expressed in at least one of IM, CM, and CMT pig groups (Table S1).

### 2.3. Gene Expression Analysis

**Figure 2:**
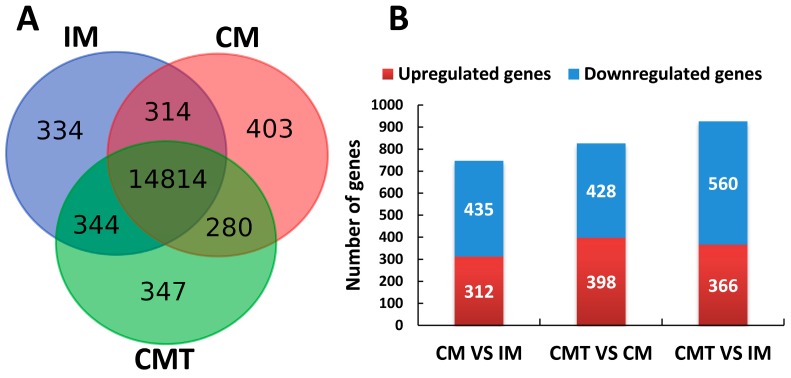
Gene expression analysis in VAT of IM, CM, and CMT pigs: (**A**) the numbers of genes expressed in IM, CM, and CMT groups; and (**B**) the numbers of DEGs between groups. IM: intact male pigs fed a HFC diet; CM: castrated male pigs fed a HFC diet; CMT: castrated pigs with testosterone replacement fed a HFC diet.

From 16,836 expressed mRNAs, 15,806, 15,811 and 15,785 expressed genes were detected in VAT of IM, CM, and CMT pig group, respectively. Among these genes, 14,814 genes were expressed in all three groups; 314, 280, and 344 genes were expressed in each pair of groups (CM versus IM, CMT versus CM, and CMT versus IM, respectively), while 334, 403, and 347 genes were exclusively found in IM, CM, and CMT pigs, respectively (Figure 2A and Table S1).

### 2.4. Identification of Differentially Expressed Genes (DEGs)

Based on our criteria of defining DEGs, 747 (312 upregulated and 435 downregulated), 826 (398 upregulated and 428 downregulated), and 926 (366 upregulated and 560 downregulated) DEGs were identified between groups of CM versus IM, CMT versus CM, and CMT versus IM, respectively (Figure 2B and Table S2). Totally, 1732 DEGs between any two groups were found in the study (Table S2).

### 2.5. Gene Ontology (GO) Enrichment Analysis of DEGs between Different Groups

**Figure 3:**
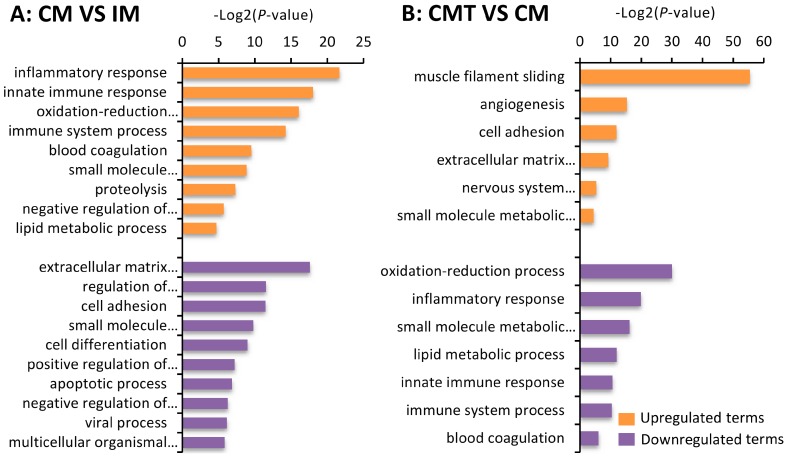
Gene ontology (GO) analysis of differentially expressed genes (DEGs) between groups: (**A**) GO terms associated with the DEGs between CM versus IM; and (**B**) GO terms associated with the DEGs between CMT versus CM. IM: intact male pigs fed a HFC diet; CM: castrated male pigs fed a HFC diet; CMT: castrated pigs with testosterone replacement fed a HFC diet.

To obtain the biological functions of DEGs in VAT of IM, CM, and CMT pigs, Gene Ontology (GO) enrichment analysis was further performed. The results showed that significantly upregulated GO terms between CM and IM pigs were mainly enriched in inflammatory response, innate immune response, and oxidation-reduction, while downregulated DEGs were mainly involved in extracellular matrix organization, regulation of transcription from RNA polymerase II promoter, and cell adhesion (Figure 3A and Table S3). Furthermore, upregulated DEGs between CMT and CM pigs were mainly enriched in muscle filament sliding, angiogenesis, and cell adhesion, while downregulated DEGs were mainly involved in oxidation-reduction process, inflammatory response, and lipid metabolic process (Figure 3B and Table S4).

### 2.6. Series-Cluster Analysis of DEGs

**Figure 4:**
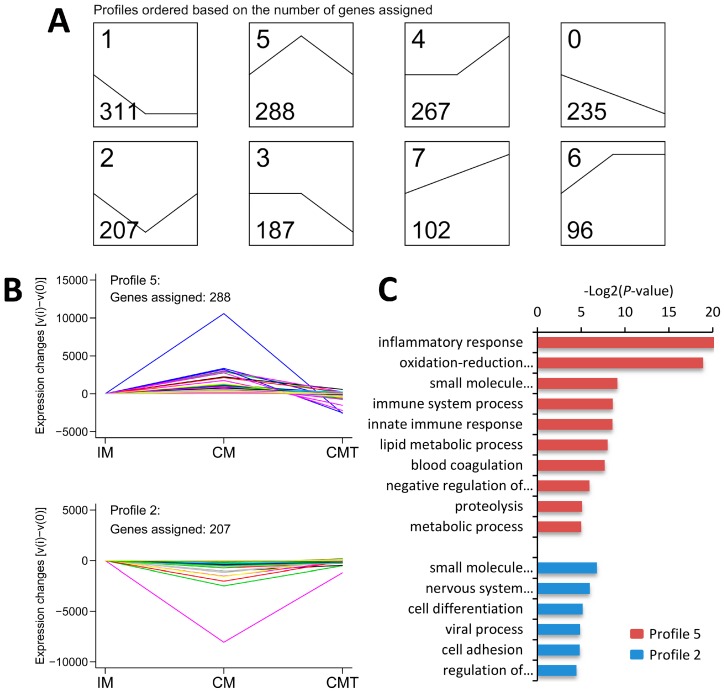
Gene expression tendencies and gene ontology (GO) analysis: (**A**) series-cluster analysis for gene expression profiles of DEGs; (**B**) gene expression tendencies of profile 5 and 2. Profile 5 included 288 DEGs that increased in CM pigs but decreased in CMT pigs compared with IM pigs, while profile 2 included 207 DEGs that decreased in CM pigs but increased in CMT pigs compared with IM pigs; and (**C**) Enriched GO terms in profiles 5 and 2. IM: intact male pigs fed a HFC diet; CM: castrated male pigs fed a HFC diet; CMT: castrated pigs with testosterone replacement fed a HFC diet.

To further refine the expression modes of DEGs in all groups, after excluding 39 DEGs with 0 value in each group, 1693 out of 1732 DEGs were used for series-cluster analysis, which were categorized into eight clusters (Figure 4A). Two of the clusters, named profiles 5 and 2, were the representative profiles for discovering the common changes induced by testosterone deficiency (Figure 4A). Compared with IM pigs, profile 5 contained 288 genes that were characterized by increased expression in CM pigs but decreased in CMT pigs, while profile 2 contained 207 genes that were characterized by decreased expression in CM pigs but increased in CMT pigs (Figure 4B and Table S5).

### 2.7. GO Enrichment Analysis of DEGs Involved in Profile 5 and 2

**Figure 5:**
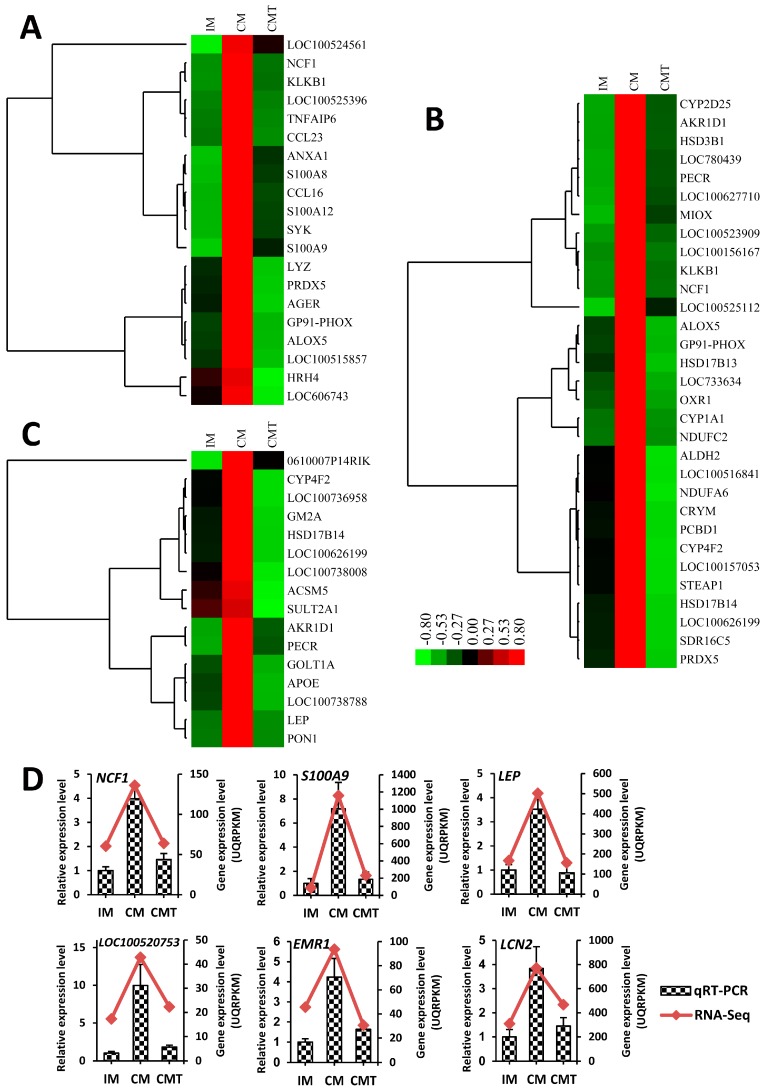
DEGs involved in inflammatory response, oxidation-reduction process and lipid metabolic process: (**A**) heat map for genes involved in inflammatory response; (**B**) heat map for genes involved in oxidation-reduction process; (**C**) heat map for genes involved in lipid metabolic process; and (**D**) expression levels of six genes (*NCF1*, *S100A9*, *LEP*, *LOC100520753* (*CD68*), *EMR1*, and *LCN2*) detected by qRT-PCR that were in agreement with the RNA-Seq results. Data were expressed as means ± SEMs, *n* = 4 per group. IM: intact male pigs fed a HFC diet; CM: castrated male pigs fed a HFC diet; CMT: castrated pigs with testosterone replacement fed a HFC diet.

**Figure 6:**
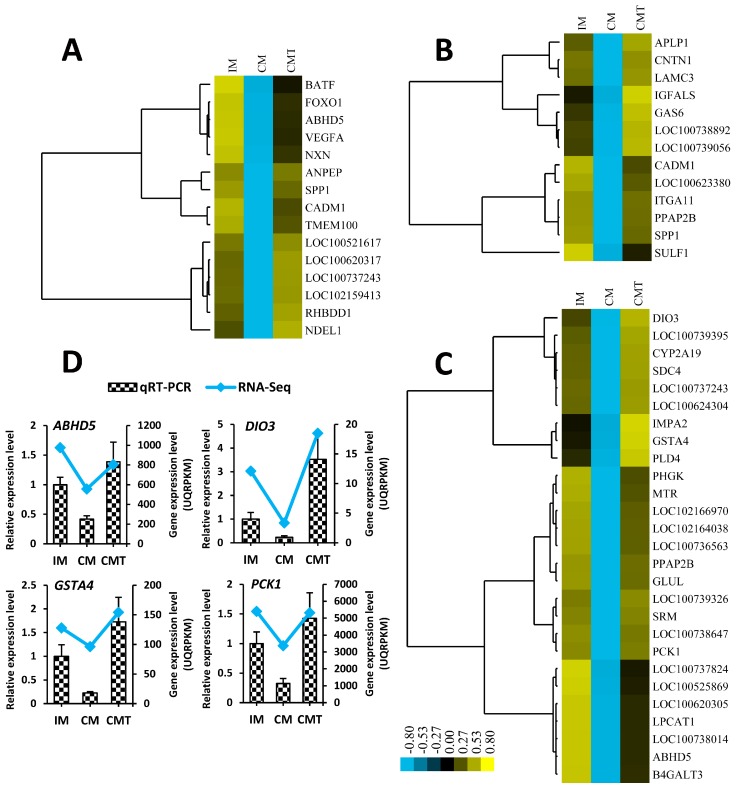
DEGs involved in cell differentiation, cell adhesion and small molecule metabolic process: (**A**) heat map for genes involved in cell differentiation; (**B**) heat map for genes involved in cell adhesion; (**C**) heat map for genes involved in small molecule metabolic process; and (**D**) expression levels of four genes (*ABHD5*, *DIO3*, *GSTA4*, and *PCK1*) detected by qRT-PCR that were in agreement with the RNA-Seq results. Data were expressed as means ± SEMs, *n* = 4 per group. IM: intact male pigs fed a HFC diet; CM: castrated male pigs fed a HFC diet; CMT: castrated pigs with testosterone replacement fed a HFC diet.

As shown in Figure 4C and Figure 5A–C, and Table S6, GO terms of DEGs involved in profile 5 were mainly enriched in inflammatory response, oxidation-reduction process, immune system process, and lipid metabolic process, while GO terms of DEGs involved in profile 2 were mainly involved in small molecule metabolic process, cell differentiation, and cell adhesion (Figure 4C and Figure 6A–C, and Table S7). To verify the mRNA expression of genes related with these above processes, quantitative real-time RT-PCR (qRT-PCR) was performed using RNA samples from IM, CM, and CMT groups applied for RNA-Seq. Ten genes, including six genes (*NCF1*, *S100A9*, *LEP*, *LOC100520753* (*CD68*), *EMR1*, and *LCN2*) related with inflammatory response, oxidation-reduction process and lipid metabolic process from profile 5 and four genes (*ABDH5*, *DIO3*, *GSTA4*, and *PCK1*) related of cell differentiation and small molecule metabolic process from profile 2, were selected for qRT-PCR verification. Consistent with RNA-Seq data, the expression of six genes from profile 5 were increased in CM pigs but decreased in CMT pigs (Figure 5D), while four genes from profile 2 were decreased in CM pigs but increased in CMT pigs (Figure 6D), indicating the strong consistence between RNA-Seq and qRT-PCR data.

## 3. Discussion

Generally, testosterone deficiency or HFC diet resulted in increased VAT accumulation. With aging or hypogonadal of obese individuals, these two factors act together on VAT deposition. However, the role of testosterone in regulating VAT accumulation under HFC diet is still largely unclear. In this study, our pig model fed a HFC diet provides a great opportunity for discovering the role of testosterone deficiency for VAT changes under HFC diet-induced state. Our data found VAT accumulation was increased in CM pigs but decreased in CMT pigs (Figure 1A), indicating testosterone deficiency significantly exacerbated VAT accumulation. In addition, this VAT accumulation process can be reversed by testosterone treatment, showing that testosterone replacement might be involved in the treatment strategy for abating VAT deposition caused by testosterone deficiency under HFC diet. These results are consistent with data observed from testosterone deficiency or androgen receptor knockout (ARKO) mice, which showed that castration induced abdominal obesity of HFD mice and this phenomenon could be eliminated by treating with antibiotics [20] and androgen deficiency under HFD significantly enhanced visceral and total fat accumulation of ARKO mice [21]. However, another study discovered there was no significant difference of VAT mass between obesogenic diet-fed (OGD, including greater amounts of sugars, saturated and monounsaturated fat and less protein and polyunsaturated fat compared to the standard rat chow diet) and orchiectomised (OGD + ORX) male mice [22]. Given differences in diet compositions, it is possible to change the effects on VAT deposition induced by androgen. Further investigation of roles of castration under different diet compositions is required. Additionally, except for testosterone, castration might alter the levels of other hormones, such as estrogen, inhibin and gonadotropins. However, there was no significant difference in key genes related to the levels of estrogen, inhibin and gonadotropins between any two groups, i.e., *ESR1*, *INHBA*, and *GNRH1*, and the expressions of *ESR2*, *INHA*, and *GNRHR* were undetected (Table S1), indicating that these genes or their related hormones might not have significant contributions in VAT deposition induced by testosterone deficiency under HFC diet.

Moreover, testosterone deficiency enhanced leptin levels in both serum and VAT, while testosterone treatment decreased these levels of leptin (Figure 1C and Figure 5D). As a key marker of fat deposition, leptin plays a core role of white adipose tissue (WAT) deposition [23]. In our previous study, castration increased the expression levels of *LEP* in subcutaneous and leaf fat tissue [24]. Furthermore, consistent with these data obtained from obese children and adolescents or type 2 diabetic men, which showed testosterone suppressed both leptin levels of serum and adipocytes [25,26], our current data indicated testosterone replacement resulted in significantly reduced leptin levels in serum and VAT as well as VAT deposition, further confirming the role of leptin as an indicator for VAT deposition. Alternatively, a major triglyceride-rich lipoprotein of the chylomicron, *APOE* was found to be significantly increased under testosterone deficiency. In APOE-deficient mice (*APOE*^−/−^) with diabetogenic or HFD diet, the fat mass was found to be significantly lower than that in *APOE*^+/+^ or wild-type mice, indicating that APOE-deficient might be resistant to adiposity and APOE was positively related with diet-induced adiposity [27,28]. Therefore, enhanced *APOE* under testosterone deficiency in our data might amplify castration-induced VAT deposition under HFC diet. Actually, expression levels of other well-known lipoproteins for modulating triglyceride metabolism, such as *APOA5* and *APOC3* [29,30], were also upregulated under testosterone deficiency, although the expressions of *APOC3* were not detected in IM pig groups (Table S1). In the previous study, APOE, APOC3, and APOA5 were shown to be positively associated with plasma triglyceride levels [31,32], which indicated VAT accumulation. Accordingly, our previous results found the levels of serum triglyceride were highly significant increased under testosterone deficiency in pigs fed a HFC diet [18]. Thus, our data suggested that these enhanced lipoproteins might contribute to VAT changes caused by testosterone deficiency under HFC feeding. Interestingly, we did not find any significant difference in other common adipogenic markers, such as *ADIPOQ*, *PPARγ*, and *CEBPα*, suggesting that these genes might not have significant roles in the regulation of castration-induced VAT deposition under HFC feeding.

As expected, testosterone deficiency with HFC induced the expression of genes related with inflammatory response. The marked genes, such as *LOC100520753* (*CD68*), *EMR1*, *LCN2*, *S100A8*, *S100A9*, and *S100A12* (Figure 5 and Table S5), were upregulated in CM pigs but downregulated in CMT pigs, suggesting testosterone replacement in castrated pigs might decrease the risk of inflammation. CD68 and EMR1 are two key markers of macrophage infiltration in WAT. In previous studies, the expressions of *CD68* and the extent of adiposity and macrophage infiltration in obese mice were increased under Zn deficiency [33]; CD68+ cell percentage as well as *CD68* mRNA expressions were positively related with VAT area in women [34]; percentage of cell expressing EMR1 (F4/80) was positively correlated with adipocyte size and macrophage accumulation in obese mice [35]; and the expression levels of *EMR1* were significantly reduced in the cocoa-supplemented HFD mice with decreased retroperitoneal WAT weights compared with HFD-fed mice only [36], showing that *CD68* and *EMR1* could play important roles on WAT accumulation and macrophage infiltration under adiposity. In this study, *CD68* and *EMR1* were significantly increased in testosterone deficiency of CM pigs and decreased in testosterone replacement of CMT pigs, suggesting that testosterone deficiency with HFC might exacerbate macrophage infiltration in VAT. Furthermore, *LCN2*, an inflammation-pathway related factor, was discovered to be regulated by testosterone deficiency. Actually, the levels of *LCN2* mRNA were dramatically increased in adipose tissue of obese mice compared with that of lean individuals [37] and were positively correlated with pro-inflammatory markers including *CD68* in VAT of obese patients [38]. Therefore, our results suggested that enhanced *LCN2* in VAT might contribute to inflammation under testosterone deficiency. Additionally, other important inflammation related markers, such as *S100A8*, *S100A9*, and *S100A12*, were also induced by testosterone changes. Our data are consistent with those observed by Sekimoto et al (2012) and Yamaoka et al (2013) [39,40], who found the expression levels of *S100A8*, *S100A9*, and *S100A12* were positively correlated with VAT adiposity in human and overexpression of *S100A8* and *S100A9* could increase adipose inflammation. Most recently, upregulated *S100A8* expression levels were shown to contribute to the very early stage of pathogenesis of obesity and induce local inflammation [41]. Obviously, our data strongly supported that testosterone deficiency with HFC might enhance the risk of inflammation via upregulating the expression levels of inflammatory markers.

Furthermore, we found the genes involved in oxidation-reduction process were induced by testosterone deficiency with HFC and resulted in up-regulation of oxidant-related genes (i.e., *NCF1* (*p47phox*) and *NOX2* (*GP91-PHOX*)) and down-regulation of antioxidant-related genes (i.e., *GPX3* and *GSTA4*) (Figure 5 and Figure 6, and Table S5). It is not surprising because oxidative related process such as oxidative stress is associated with adiposity caused by VAT accumulation [42,43]. In fact, as two important oxidant genes of oxidative stress, the expression levels of *p47phox* and GP91-PHOX were shown to be positively associated with VAT mass in mice or rats [43,44], suggesting VAT changes resulted in oxidative stress by regulating the expression of *p47phox* and GP91-PHOX. For antioxidant markers, oxidative stress reduced the levels of *GPX3* in the plasma of obese subjects and adipose tissue of HFD mice [45,46] as well as *GSTA4* expression in adipose tissue of obese C57BL/6J mice [47]. Therefore, these data demonstrated that fat deposition might enhance oxidative stress by upregulating oxidant-related genes and downregulating antioxidant-related genes. Consistent with these data, our results supported that VAT deposition of testosterone deficiency with HFC in pigs is associated with oxidative related process by regulating oxidant- and antioxidant-related genes.

In addition, testosterone deficiency with HFC mainly decreased the genes involved in cell differentiation, cell adhesion and small molecule metabolic process. Some important genes, i.e., *ABHD5* for cell differentiation, *SPP1* for both cell differentiation and cell adhesion, *GAS6* for cell adhesion, and *DIO3* and *PCK1* for small molecule metabolic process (Figure 6 and Table S5), were significantly downregulated under testosterone deficiency with HFC. ABHD5, also known as CGI-58, is a master regulator of triacylglycerol hydrolysis [48] and its knockdown resulted in abnormal accumulation of lipid droplets in 3T3-L1 preadipocytes [49]. In HFD-induced mice, CGI-58 deficiency aggravated macrophage inflammation [50], but Knockdown of CGI-58 was found to prevent HFD-induced obesity and reduce the expression of both lipolytic and lipogenic genes in WAT [51], indicating that the role of *CGI-58* for obesity under HFD is still controversial. In this study, *ABHD5* was significantly downregulated in CM pigs and testosterone replacement significantly increased its expression in CMT pigs, providing new evidence that *ABHD5* might have roles of VAT accumulation regulated by testosterone deficiency with HFC. SPP1, also known as osteopontin, a secreted extracellular matrix protein that binds to specific integrins to promote cell adhesion in porcine trophectoderm cell [52], had been shown to induce brown adipogenesis from white preadipocytes [53]. Moreover, the expression of SPP1 in principal cells of the rat epididymis disappeared after orchiectomy and can be restored with testosterone replacement [54], demonstrating that testosterone could positively regulate SPP1 expression. GAS6, a vitamin K-dependent protein, was shown to regulate cell adhesion in different cells such as endothelial cells and schwannoma cells [55,56]. Furthermore, mutations of *GAS6* were shown to be associated with obesity and type 2 diabetes individuals [57] and its serum levels were positively associated with testosterone levels in male patients with coronary heart disease [58], and androgen could directly regulate *GAS6* expression by binding the androgen-response elements of its promoter regions for inhibition of vascular calcification [59]. In the present study, downregulated *GAS6* and *SPP1* under testosterone deficiency with HFC were discovered, further confirmed the role of androgen signaling to regulate these two genes in diet-induced VAT deposition. *DIO3*, a master gene of heat production and energy metabolism, was found to be associated with multiple traits of fat deposition in pig [60], but so far, the role of *DIO3* in VAT deposition induced by androgen is unclear. Here our results provide a novel clue that *DIO3* might play an important role in VAT deposition under testosterone deficiency. *PCK1*, a rate-limiting enzyme for encoding the gluconeogenesis [61], was downregulated by testosterone deficiency with HFC. Consistent with those data observed by Niang et al (2011) [62], who found leptin can significantly downregulated the mRNA expressions of *PCK1* in rat WAT, our data also discovered the expression levels of *PCK1* were significantly decreased with the increase of leptin levels under testosterone deficiency with HFC, showing that the function of *PCK1* in VAT deposition might be induced by leptin signaling. Accordingly, our results indicated that testosterone deficiency with HFC might reduce cell differentiation, cell adhesion and small molecule metabolic process by down-regulating their related genes. However, some upregulated genes involved in small molecule metabolic process were simultaneously found in this study, hence the role of testosterone deficiency with HFC in small molecule metabolic process need to be investigated further. Taken together, our data provide a comprehensive clue on the role of androgen in cell differentiation, cell adhesion, and small molecule metabolic process.

## 4. Materials and Methods

### 4.1. Animals

Chinese Wuzhishan miniature male pigs (6–7 months old) were obtained from the Institute of Animal Sciences, Hainan Academy of Agricultural Sciences (Haikou, China). The animals received a standard diet without cholesterol during a 7-week “pretreatment period” for acclimation to the environment. At week 7, the pigs were divided into three groups as follows: intact male pigs fed a HFC diet (IM), castrated male pigs fed a HFC diet (CM), and castrated pigs with testosterone replacement fed a HFC diet (CMT). Among them, IM pigs received only skin incisions which were closed by suture, CM pigs were gonadectomized through two incisions in the perineum which were closed by suture, and CMT pigs were given testosterone treatment after castration by intramuscular weekly injection with testosterone propionate (10 mg/kg body weight; Sigma-Aldrich, St. Louis, MO, USA) dissolved in corn oil. All surgical operations were performed under anesthesia. The HFC diet was fed for 12 weeks starting from week 8. The HFC diet was comprised of 73% normal swine diet, 15% lard, 10% egg yolk power, 1.5% cholesterol, and 0.5% sodium cholate. All experimental procedures were approved by the Institutional Animal Care and Use Committee of the Zhejiang Chinese Medical University (2013450; 9 December 2013).

At the end of the experimental period, animals were weighted and killed by exsanguination under sodium pentobarbital anesthesia. Blood samples were collected and centrifuged at 3000× *g* at 4 °C for 15 min and then stored at −80 °C for serum measurement. VAT of each animal was derived from retroperitoneal adipose tissue and weighed, followed by freezing storage in liquid nitrogen for RNA extraction. VAT fat was measured as a ratio of VAT weight refers to the proportion of body weight. Serum testosterone concentrations were measured using an RIA kit (B10B, Beijing North Institute of Biological Technology, Beijing, China), and serum leptin levels were measured using a commercially available kit (50R-E.1511P, Tianjin Anoric Bio-technology, Co., Ltd., Tianjin, China) and analyzed by an Automatic Biochemistry Analyzer (Hitachi 7020, Tokyo, Japan). The intra-assay and inter-assay coefficients of variation were below 10% and 15% for testosterone, respectively, and the inter-assay coefficients of variation were below 6% for leptin. The assay sensitivities were 0.02 ng/mL and 0.1 ng/L for testosterone and leptin, respectively.

### 4.2. RNA Extraction, cDNA Library Construction and Ion Proton Sequencing

Total RNA was extracted using TRIzol reagent (Invitrogen, Carlsbad, CA, USA) according to the manufacturer’s protocol. RNA integrities and qualities were assessed by Bioanalyzer 2100 (Agilent Technologies, Santa Clara, CA, USA) and agarose gel electrophoresis. The RNA integrity number (RIN) of all samples was more than 7.0. Then, RNA samples from IM, CM, and CMT groups (*n* = 4 per group) were pooled with equal quantities, respectively, and were used for cDNA library construction and Ion-proton sequencing. Briefly, cDNA libraries for single-end sequencing were prepared using the Ion Total RNA-Seq Kit v2.0 (Life Technologies, Carlsbad, CA, USA) according to the manufacturer’s instructions. After diluting and mixing of samples, the mixture was processed on OneTouch 2 instrument and was enriched on OneTouch 2 ES station (Life Technologies, Carlsbad, CA, USA). Finally, the enriched mixture samples were loaded on to 1 P1v2 Proton Chip and sequenced on Proton Sequencers according to the Ion PI Sequencing 200 Kit v2.0 (Life Technologies, Carlsbad, CA, USA).

### 4.3. Sequence Mapping and DEGs Filtering

Raw sequencing data were filtered by removing the adaptor sequences, reads with >5% ambiguous bases, and low-quality reads with qualities <13. These clean data were then mapped to the current pig reference genome (Sscrofa 10.2) using the MapSplice program [63]. UQRPKM values [64,65] were used to measure the expression levels of each gene. DEGSeq algorithm of R package was used to identify DEGs [66]. The DEGs were defined as fold changes of 1.5 or more, *p* value less 0.05 after false discovery rate (FDR) correction, and UQRPKM values greater than or equal to 1 in any two groups.

### 4.4. Cluster Analysis

Hierarchical clustering analysis was performed using Cluster 3.0 and was visualized using the Java Treeview (version 1.1.6r2, Stanford University, Stanford, CA, USA). The expression profiles of DEGs were determined by series-cluster analysis based on the Short Time-Series Expression Miner (STEM) method [67].

### 4.5. Gene Ontology (GO) Analysis

Gene ontology (GO) analysis of DEGs using GO annotation from the Gene Ontology (http://www.geneontology.org/). Fisher’s exact test was applied to identify the significant GO categories. Terms with *p* values less than 0.05 and the number of matching genes in term (DifGene) more than 5% of all used genes (AllDifGene) were selected as significant or enriched terms.

### 4.6. Quantitative Real-Time RT-PCR

Ten genes (six from profile 5 and four from profile 2) were analyzed by quantitative real-time RT-PCR (qRT-PCR) for validating the data from RNA sequencing. The primers of each gene are listed in Table S8. Approximately 1 μg RNA was reverse transcribed using an MMLV-RT Kit (Promega, Madison, WI, USA) according to the manufacturer’s protocol. Next, qRT-PCR was performed using a standard SYBR Green PCR kit (Takara, Dalian, China) and was processed on StepOnePlus™ Real-Time PCR Detection System (Applied Biosystems, Foster City, CA, USA). The qRT-PCR conditions were used as follows: 95 °C for 5 min, followed by 40 cycles of 95 °C for 15 s, 60 °C for 30 s, and 72 °C for 30 s. β-Actin was used as an internal control to normalize gene expression and assays were run in triplicate. The 2^−ΔΔ*C*t^ method was used to determine the expression level differences [68].

### 4.7. Statistical Analysis

Statistical analyses were performed using SPSS v17.0 software (SPSS, Chicago, IL, USA) and the results showed as means ± SEMs. Statistical differences between groups were examined using ANOVA followed Bonferroni multiple comparison, and *p* values of less than 0.05 and 0.01 were considered as significant and highly significant, respectively.

### 4.8. Data Accessibility

The datasets have been submitted to the Gene Expression Omnibus (GEO) database of NCBI (accession number GSE77544).

## 5. Conclusions

In summary, a genome-wide view of transcriptome changes of VAT with HFC induced by testosterone deficiency in miniature pig model was investigated here. Our data discovered that testosterone deficiency in pigs fed a HFC diet mainly increased the changes of genes associated with inflammatory process, oxidation-reduction process, and lipid metabolic process, while reduced other genes mainly involved in cell differentiation and cell adhesion. Furthermore, this study provides novel insights for comprehensively understanding the mechanisms of the VAT changes induced by testosterone deficiency, revealing possible clues for treatment or abatement of visceral adiposity under testosterone deficiency.

## Figures and Tables

**Table 1:** Statistics of the sequencing reads mapping to the reference Sscrofa 10.2 genome.

Terms	IM	CM	CMT
All reads	26,818,106	24,658,796	23,870,297
Un mapped reads	4,906,529	4,295,752	4,089,813
Mapped reads	21,911,577	20,363,044	19,780,484
Mapping rate	0.817	0.826	0.829
Unique mapping	20,705,544	19,206,414	18,686,768
Unique mapping rate	0.772	0.779	0.783
Repeat mapping	1,206,744	1,157,303	1,094,352

IM: intact male pigs fed a high-fat and high-cholesterol (HFC) diet; CM: castrated male pigs fed a HFC diet; CMT: castrated pigs with testosterone replacement fed a HFC diet.

## Supplementary Materials

Supplementary materials can be found at www.mdpi.com/1422-0067/17/12/2125/s1.
